# The Emergence of Lying for Reputational Concerns in 5-Year-Olds

**DOI:** 10.3389/fpsyg.2021.700695

**Published:** 2021-09-22

**Authors:** Mareike Klafka, Ulf Liszkowski

**Affiliations:** Department of Psychology and Human Movement Science, Developmental Psychology, Institute for Psychology, University of Hamburg, Hamburg, Germany

**Keywords:** lying, reputation management, group reputation, prosocial behavior, cooperation

## Abstract

Research suggests that even young children engage in strategic behaviors to manipulate the impressions others form of them and that they manage their reputation in order to cooperate with others. The current study investigated whether young children also lie in order to manage their, or their group’s, reputation in front of ingroup and outgroup members. Five-year old children (*n*=55) were randomly assigned to an individual reputation condition or a group reputation condition. Then, they played a mini dictator game in which they could share privately any number of their or their group’s stickers with an anonymous child. Participants then met ingroup and outgroup members, established through a minimal group design, *via* a pre-recorded, staged Skype call. Group members asked the participant how many stickers she, or her group, had donated. Results revealed that children stated to peers to have donated more than their actual donation, with no differences between conditions and no difference toward ingroup and outgroup members. Findings suggest that by 5years of age, children use lying as a strategy to manage their reputation.

## Introduction

### Reputation Management in Childhood

Young children engage in strategic behaviors to manipulate the impressions others form of them. The current study is a first step in investigating whether children also use lying as a strategic behavior to manage their reputation, for example when being asked by peers about their generosity. While a host of studies has investigated children’s lying for selfish and prosocial reasons (Talwar and Crossman, 2011), little is known about children’s use of lying as a strategy to manage their reputation.

Research suggests that 5-year old children behave more prosocially in the presence of others (Engelmann and Rapp, 2018). They share more and steal less when they are being watched by a peer than when they are alone (Engelmann et al., 2012; Yazdi et al., 2020). They are less likely to cheat in a guessing game, if they are told that they have a positive reputation to maintain, even if nobody is watching them and if not to cheat conflicts with their personal interest (Fu et al., 2016b). In contrast, telling children that they have a reputation for being smart results in more lying in 3–5-year-olds in a guessing game (Zhao et al., 2018). This suggest that even 3-year old children are responsive to reputational cues in their morally relevant behavior. With age, children start to explicitly reason about intentions and social outcomes. By 8years of age, they are more generous in a sharing game not only when their behavior is observed by a third party, but also when it can affect their chances of being chosen for a subsequent game (Herrmann et al., 2019). Moreover, 8-year-olds begin to understand ulterior motives. They reason that children who offer gifts to others in public compared to private settings might have an ulterior motive to enhance their reputation (Heyman et al., 2014). Further, they understand that excessively promoting one’s own good deeds can have negative reputational consequences, and show modesty to manage their reputation by falsely denying their good deeds (Fu et al., 2016a). These modesty-related lies seem to be particularly influenced by cultural socialization. In sum, findings suggest that, from an early age, children manage their reputation in order to be socially accepted and to cooperate with others (Engelmann and Rapp, 2018; Herrmann et al., 2019).

The emergence of lying as a strategy to manage reputational concerns is still poorly understood. Do young children overstate their prosociality in order to manage their reputation and to cooperate with others? On the one hand, children’s awareness of moral standards may prevent them from telling reputational lies. Research has revealed that from age 4 on children generally evaluate lies worse than telling the truth and they expect greater self-approval for truth-telling and greater self-disapproval for lying (Bussey, 1999). Thus, reputational lying may conflict with their moral (self-)evaluation. On the other hand, children may expect greater other-approval when lying to manage their reputation. Moreover, findings from Bussey (1999) suggest that they take motivational factors into consideration when morally evaluating lies. For instance, they expect greater negative evaluative reactions for antisocial than for prosocial lies. The current study thus asks whether young children begin to make deliberately untrue statements for reputational concerns. The current study is the first to examine whether lying for reputational concerns is present at the age when children begin to engage in other strategic behaviors to manage their reputation, around 5years of age.

### Group Biases in Reputation Management

Understanding the scope of reputation management at its incipient emergence contributes to the broader endeavor of understanding the nature and origins of human social cooperation. A characteristic adaptation for human social cooperation and cultural life is the ability to take the group’s perspective and to care about the group (Tomasello, 2019). Research suggests that children show loyalty to their group from early on. For instance, they are less likely to reveal the secret of an ingroup compared to an outgroup member (Misch et al., 2016) and they are less likely to blow the whistle on their ingroup than on the outgroup (Misch et al., 2018). Young children are loyal to their group, even if it comes with personal costs. With age, children become more likely to tell lies to benefit the collective (Fu et al., 2008). Some research suggests that 5-year-olds have selective reputational concerns with ingroup and outgroup members and care more about their reputation with potential reciprocators, than with individuals with whom they would not later interact. For instance, children share more resources with an anonymous recipient, if they are watched by an ingroup rather than an outgroup member (Engelmann et al., 2013).

Further research has revealed that children exhibit an ingroup bias when distributing a resource by sharing more with an ingroup member than with an outgroup member, and they evaluate ingroup sharing as nicer than outgroup sharing (Yazdi et al., 2020). Interestingly, findings suggest that merely the perception of belonging to different social groups leads to ingroup favoritism and outgroup discrimination when distributing a resource (Tajfel et al., 1971). This effect has been replicated in numerous studies, even if intergroup categorization was based on irrelevant classification criteria such as aesthetic preference for Klee’s or Kandinsky’s paintings (minimal group paradigm). Effects are also well established with children where intergroup categorization is based on criteria such as wearing t-shirts of the same color (Dunham et al., 2011). Findings suggest that ingroup favoritism may be caused by ingroup reciprocity and outgroup fear (Gaertner and Insko, 2000) and that cooperative behavior toward ingroup members may be attractive because it gives access to a generalized exchange system in the group (Yamagishi and Mifune, 2008). Ingroup favoritism may serve as a reputation mechanism, for example, the presence of an eye-painting enhances resource allocation (Mifune et al., 2010). While there is much research on ingroup favoritism in the context of resource allocations, much less is known about ingroup and outgroup biases in the context of reputational lies. Beyond this, it has remained unexplored whether young children will also manage their group’s reputation and lie about their group’s generosity to peers. Accordingly, the current study investigated, whether children may lie for reputational concerns for themselves, or their ingroup, and perhaps more or less toward an ingroup or outgroup member.

### The Current Study

The current study provides a first step in investigating the emergence of lying for reputational concerns in 5-year-olds and to explore potential group biases. Participants played a mini dictator game (Rapp et al., 2019) in which they could share all, none or any number of their, or their group’s, stickers with an anonymous, hypothetical child. In the individual reputation condition, children donated individually, whereas in the group reputation condition children donated on behalf of a group consensus.

Intergroup categorization was based on minimal group markers (i.e., wearing a blue or a red cap; Dunham et al., 2011; Engelmann et al., 2013). In both conditions, the ingroup and outgroup members were revealed to the children *via* a pre-recorded Skype call. After the donation game, an ingroup and an outgroup member called separately with the first child counterbalanced across participants. Both children asked the participant how many stickers she, or the group, had donated. We compared statistically the participant’s answers to their actual number of donated stickers. A background assumption born from previous research was that children would distribute the stickers in their favor (Smith et al., 2013). To establish that children indeed had a normative expectation about how many stickers one ought to donate in public – which should put them in conflict once they had to publicly announce their own donation – we asked children in a separate task to decide how many stickers a hypothetical character should publicly donate to another anonymous child. We compared statistically the participant’s answers to their actual number of donated stickers and their stated number of stickers. The task thus contrasted participants’ distributions and their sense of fairness. In line with previous research, we expected children to donate less than they perceived as fair.

If lying for reputational concerns emerges at the age of 5years, the number of stickers they stated to have donated should exceed the number of donated stickers. If 5-year-olds use lying in order to manage their group’s reputation, they should lie about the amount of donated stickers also in the group reputation condition, and the number of stated stickers should exceed the number of donated stickers. Since the group’s reputation indirectly also refers to each group member individually, children may be concerned with both, their individual and their group’s reputation equally. Thus, we did not expect *a priori* any differences between lying for the individual or for the group’s reputation.

If children experience group biases when managing their reputation, answers to the ingroup and outgroup member could differ. In line with previous research, one could expect that children would be more prone to lie to protect their reputation (individually or group-based) in the eye of an ingroup member than an outgroup member. On the other hand, from an evolutionary perspective, they may both serve as potential partners for social cooperation in the long term and children may be concerned with their reputation with an ingroup and outgroup member equally. For example, like showing physical strength, showing social integrity may advocate one’s fitness or superiority toward anyone, including outgroup members.

Finally, if children manage their reputation, they should have normative expectations about how one ought to share. Thus, the mean number of normatively expected shared stickers in the hypothetical scenario should be greater than the actual number of donated stickers, and likely not be different from the stated number of donated stickers.

## Materials and Methods

### Participants

In total, 55 Caucasian 5-year-olds participated in this study (Mean age=65.58months, *SD*=3.75). Participants were recruited from the department’s database of parents who had agreed to participate in child studies. Twenty-seven children were included in the individual reputation condition (13 girls) and 28 children were included in the group reputation condition (13 girls). Eighteen children chose to be in the red group and 16 to be in the blue group with an equal distribution across gender.

### Design and Procedure

This study was non-invasive and conducted according to the national ethics guidelines. The general procedure was approved by the local ethics committee of the authors’ institution. Before the beginning of the experiment, parents gave written informed consent. After a short warm-up with the experimenter, children were randomly assigned to one of two between-subjects conditions: The individual reputation condition or the group reputation condition. In both conditions, the child could choose between becoming a member of the blue or the red group by choosing to wear a blue or red cap (minimal group paradigm).

**Figure 1:**
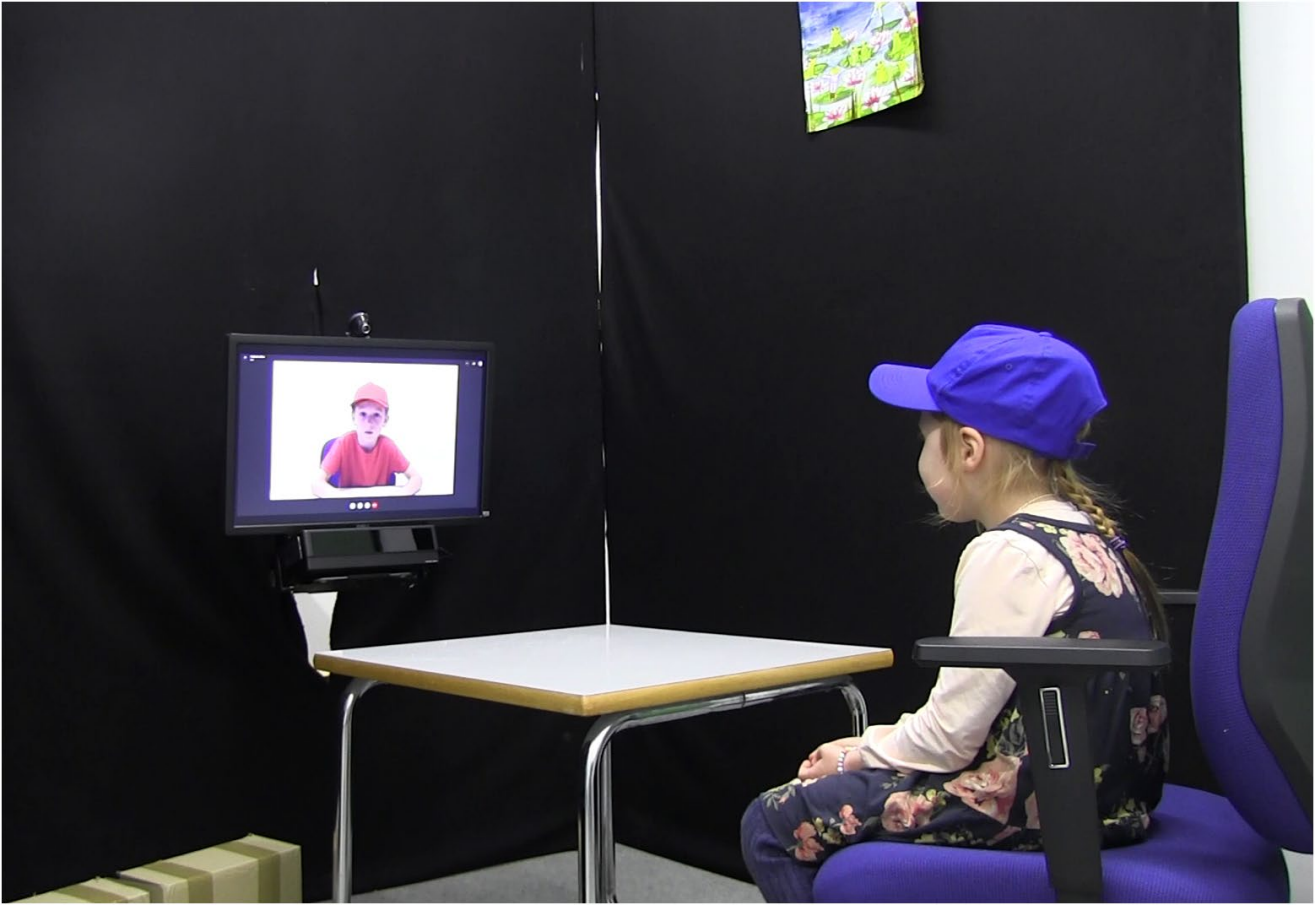
Setup during the individual skype calls (test questions).

Next, the experimenter presented a blue (red) bag (matching the participant’s group), and a gray bag to the participant and explained that the blue or red bag and everything it contains belonged to the participant, or her group, depending on the condition, and that the gray bag and everything it contains belonged to another child. In both conditions, participants then played a mini dictator game (Rapp et al., 2019). The participant received 10 small yellow plastic containers and the experimenter explained that each container contained a sticker and that they could keep them and take them all home (in the group condition: they could keep them together with their group) or share some with another child who does not have any stickers. Participants did not see the actual stickers to avoid individual preferences for the stickers. Participants were told to place the (closed) containers they wanted to take home in their bag and that they could donate some of their containers to the other child by placing them in the gray bag. Participants were thus free to share all, none, or any other number of their containers. In the individual reputation condition, children donated individually whereas in the group reputation condition the group donated collaboratively. Therefore, in the group condition, an ingroup member “called” the participant *via* a pre-recorded Skype call before the allocation process started. Skype calls had been pre-recorded with four 6-year-old children (two boys, two girls) who had been invited for this purpose and had been filmed wearing red and blue caps as they repeated target sentences presented by the experimenter. The typical logo and sound signaling an incoming skype call was added to the video and replayed to the participant. The ingroup member (corresponding in cap color and gender to the participant) asked “How many stickers do we want to donate?,” and, after a pause, agreed upon the participant’s suggestion (“Ok. Then let us do that.”). Figure 1 displays the setup during the individual skype calls. It is important to note that all participants in the group condition naturally interacted with the other child, suggesting that they were not aware that this was an artificial interaction with a pre-recorded video. Then, the participant allocated the (group’s) stickers while the experimenter was out of the room. The experimenter reentered when the child had allocated the stickers and took the bags out of the room.

Next, the experimenter told the participant that four other children just played the same game in a different room. The four children were presented in a pre-recorded Skype group call, which had been produced as described above. The four videos were arranged in four quadrants of the screen as is typical for Skype group calls. The children introduced themselves in an interactive process with the experimenter to make participants believe that this was an online interaction. The experimenter greeted all children (“Oh hello, who are you?”) who then successively presented themselves (“Hey there. I am …”). The fourth child then addressed the participating child by asking “And who are you?.” After a short pause to give the participant time to answer, the experimenter asked the children “What group are you in?.” Two children, one boy and one girl, were ingroup members wearing the same cap as the participant (in the group reputation condition, one child was already known from the allocation decision). The other two children, one boy and one girl, were outgroup members wearing a cap in a different color. The two children wearing a blue cap answered simultaneously “We are in the blue group” and the two other children wearing a red cap answered simultaneously “We are in the red group.” The experimenter then said goodbye to all children and the call ended. After the call, the experimenter asked the participant about her group membership as a reminder. Then, the experimenter told the participant that an ingroup and an outgroup member (referred to as “one from your group”; “one from the other group”) would call again, because they had a question for the participant. The experimenter encouraged the participant to talk to the children and then left the room. Again, videos of pre-recorded Skype calls were displayed. In the call, the ingroup member always had the same gender as the participant, and the outgroup member had a different gender. The order of the calling children was counterbalanced.

Both children asked the participant the test question “How many stickers did you (your group) donate?.” The participant’s answers were video-recorded and transcribed. After the donation game and the Skype calls, about 5min after the actual donation, children completed a memory check. For the memory check, the experimenter opened the bags behind a barrier and asked the child if she remembered how many stickers she, or her group, had donated to the other child. This procedure made it clear for the participant that the experimenter could see the bag’s content and lying would not be an option.

Finally, participants were asked about their normative expectation of how one should distribute stickers. The experimenter told the participant an analogous, hypothetical story about a girl, Kim (for boys: about a boy Paul), illustrated with drawings. In the story, Kim got 10 candies and could share some of the candies with another child. Other children would want to know how Kim decided. The experimenter asked the participant how many candies Kim should share with the other child. It is important to note that children knew that Kim’s distribution would be made public. This way we ensured that participants did not only justify their own distribution in the dictator game, or realistically predicted a selfish distribution in an unobserved situation, but that they answered from a normative perspective.

### Analytic Strategy

Our main dependent variables were the number of donated stickers during the actual donation, the stated donation, and the normatively expected donation. Lying scores were calculated for answer to the ingroup member and for answers to the outgroup member (answer to test question minus number of donated stickers). In preliminary analyses, we first checked for effects of gender on the mean number of donated stickers, how many children donated selfishly, and how many children remembered their actual donation correctly. In our main analysis, we included only children who correctly remembered their actual donation in order to be able to distinguish genuine lying from false memory. To test for condition effects on the lying score, we ran a mixed analysis of variance. The categorical independent between-subjects variable was condition (individual choice, group choice) and the categorical independent within-subjects variable was member (ingroup member, outgroup member). The continuous dependent variable was the lying score. To test our main hypothesis of lying, we planned a directed paired *t*-test to compare the actual number of donated stickers with the stated number of donated stickers. Finally, we compared children’s actual donation to their normative expectation. Based on previous research and our predictions, we planned an *a priori* directed comparison between the number of donated stickers and children’s normatively expected donation, and tested with a paired-sample *t*-test whether children’s expected number of donated stickers would exceed their actual donation. In an exploratory analysis, we also looked at children who were in a moral dilemma, i.e., children who shared less than they perceived as fair.

## Results

### Preliminary Analyses

There were no gender differences in the amount of donated stickers [*t*(51)=0.58, *p*=0.56, *d*=0.16]. Seventy-six percent of all children distributed the stickers selfishly and donated less than five stickers. Thirty-four children (64%) passed the memory check by correctly remembering the amount of donated stickers. Sixteen percent of children did not provide a numerical answer to both, ingroup and outgroup members and were thus excluded from the omnibus analyses.

### Lying Scores Across Conditions

Since lying is different from false memories, we calculated lying scores only for children who passed the memory check. Only children who provided numerical answers to both test questions could be included (*n*=26). A 2 (condition: individual, group)×2 (member: ingroup member, outgroup member) analysis of variance for the lying scores revealed no main effect of condition [*F*(1, 24)=0.002, *p*=0.966, *η*^2^<0.001] or group [*F*(1, 24)=1, *p*=0.327, *η*^2^=0.04] and no significant interaction [*F*(1, 24)=1, *p*=0.327, *η*^2^=0.04]. Thus, data was collapsed across conditions and group for the following analysis.

### Hypothesis Tests of Lying

**Figure 2:**
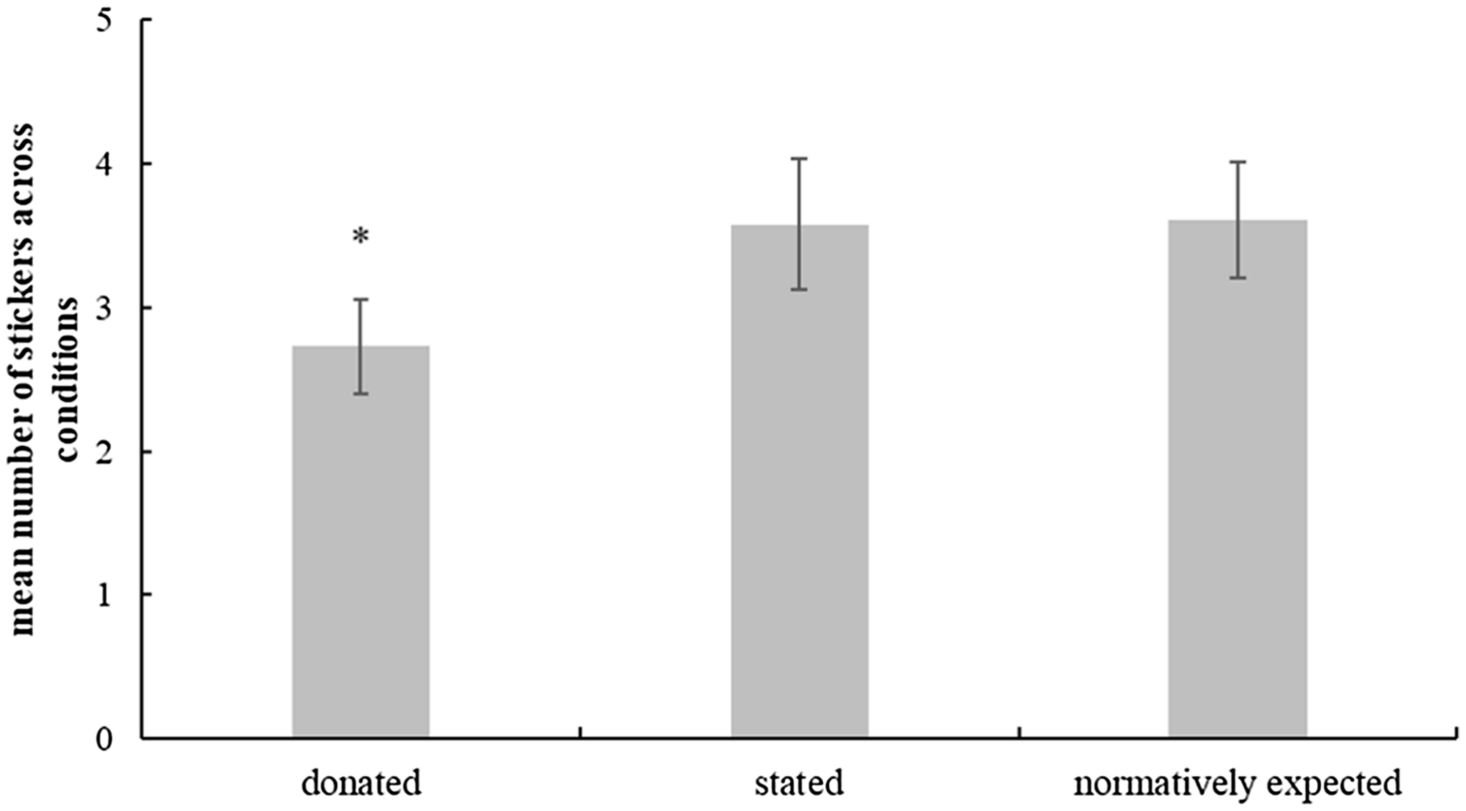
Mean number of donated stickers across conditions, mean number of stickers stated in the test-questions and normative expectation about number of donated stickers. Included were onlychildren who passed the memory check (*n*=34). Error bars depict standard error of the mean. ^*^Significantly different from both other bars, *p*<0.05.

As displayed in Figure 2, for those children who passed the memory check and who gave a numerical answer to at least one test question (*n*=30), the number they stated exceeded the number of donated stickers [*t*(29)=2.15, *p*=0.04, *d*=0.38] with an average donation of 2.73 stickers (*SD*=1.84) and a stated number of 3.58 stickers (*SD*=2.53). The pattern remained the same when all children who gave a numerical answer were included in the sample independent of their memory performance [*n*=35; *t*(34)=2.6, *p*=0.014, *d*=0.44].

### Hypothesis Tests of Normative Expectations

In the perceived norm task, participants stated that Kim/Paul should share 3.61 (*SD*=2.28) candies with the other child with no difference between conditions [*t*(51)=0.14, *p*=0.893, *d*=0.04] and no gender differences [*t*(51)=1.33, *p*=0.191, *d*=0.37]. Sixty percent of the children expected a selfish distribution and stated that Kim/Paul should share less than five candies with the other child. As predicted, the perceived norm was higher than the amount of donated stickers in the mini dictator game [*t*(32)=1.74, *p*=0.046, *d*=0.3, one-tailed, according to the directed prediction]. Exploratory analyses revealed that sixteen children were in a moral dilemma, that is, they shared less than they perceived as fair. Of those children who were in a moral dilemma, 38% had lied about their donation (not significantly different from chance, binomial test, two-tailed, *p*=0.45, *n*=16). Lying was not related to being in a moral dilemma [*φ*(35)=0.18, *p*=0.283].

## Discussion

This study investigated whether young children lie for reputational concerns. In a mini dictator game, participants could share some of their or their group’s stickers with another child. Results revealed that participants distributed the stickers selfishly, but subsequently stated to peers to have donated a higher number of stickers. This finding was independent of whether children donated the stickers individually or collaboratively as a group, and independent of whether children answered to ingroup or outgroup members. These results suggest that by 5years of age, children lie for psychological reasons, such as managing other’s impressions of them.

While the majority of children distributed the stickers selfishly in an unobserved situation, children did not behave completely selfishly. Most children (87%) shared at least one sticker with the anonymous child although they could have kept all stickers to themselves. On the one hand, this may speak for an intrinsic motivation to help others (Warneken and Tomasello, 2008). On the other hand, this does not necessarily mean that children behave altruistically, because they might still have been influenced by reputational concerns. They might have suspected to “stand to gain by being more generous than required by the rules of the game” (Sperber and Baumard, 2012, p. 501). This might have been relevant especially in the group condition, where children decided on their donation collaboratively.

When being asked about the amount of donated stickers in the test questions, children falsely claimed that they had donated about four stickers. Importantly, children did not simply err due to weak memory. First, they systematically stated a higher, not a random or lower number of stickers. Second, the memory check excluded the possibility that children had forgotten their donation. It could be argued that pretending to have shared even more than five stickers would improve one’s reputation further. However, sharing about half of the stickers might have corresponded to their fairness norm and fair partners seem to be favored in the process of partner choice for cooperation (Sperber and Baumard, 2012). Further research suggests that children are aware of the reputational costs when they make positive claims about themselves (Fu et al., 2016a; Amemiya et al., 2020). Thus, pretending to have shared more than half of the resource would not necessarily further improve their reputation.

Findings revealed no significant differences between the individual and the group condition. This is in line with the idea that the group’s reputation indirectly also refers to each group member individually. While previous research has shown an increase in children’s reputation management when their prosocial behavior is displayed publicly and compared to the behavior of other children of the group (Rapp et al., 2019), the current study did not use a competitive group setting. A direct competition between the groups might have further increased children’s identification with their group and, in particular in the group reputation condition, their lying for their group’s reputation. It can be discussed whether participants perceived the collaborative donation differently from the individual donation. In the group condition, the child in the Video always agreed upon the participant’s suggestion. Thus, we cannot rule out the possibility that children in the group condition may have felt that they were deciding alone. A potential solution would be to use a confederate child for the collaborative decision. On the other hand, the activation of social membership in the beginning of the experiment (minimal group markers) might as well have led children in the individual condition to act more as group members than just individuals. It should be clarified, however, that children’s interactions during the Skype calls and their behavior during the dictator game did not provide any evidence for such possible confound.

Findings of the current study did not reveal any differences between reputational lying with members of the ingroup or outgroup. Previous research has revealed that children show ingroup favoritism when distributing a resource (Dunham et al., 2011) and that they share more with an anonymous child, when they are observed by an ingroup than by an outgroup member (Engelmann et al., 2013). Group biases in the context of reputational lying are still poorly understood. The absence of evidence might be caused by the relatively small sample size. Furthermore, the current study did not include a direct cooperation or reciprocity between the participant and the other children, and the test questions were asked *via* prerecorded videos rather than in a direct interaction. Yet, research suggests that children behave more generously, if their behavior can affect their chances of being chosen for a game (Herrmann et al., 2019) or if the child watching them can reciprocate later (Engelmann et al., 2013). The absence of direct cooperation or reciprocity might thus have resulted in a comparatively small identification with the ingroup compared to the outgroup and only a small incentive to lie in the current study. It should be mentioned, however, that all children naturally interacted with the confederates in the video suggesting a certain similarity between the artificial and natural interactions. On the other hand, from an evolutionary perspective, potential cooperative partners may be appreciated independent of whether they initially belonged to the ingroup or outgroup. Thus, children may be concerned with their reputation with ingroup and outgroup members equally.

Children normatively expected one should provide a higher donation than they provided privately. This finding is consistent with that of other studies reporting a gap between children’s fairness judgments and their actual behavior (Smith et al., 2013). Accordingly, even 3-year-olds know the norm of equal sharing but often do not act in accordance with this norm. They favor themselves when sharing a resource even though they state that they should share equally. Thus, before age 8, children understand the norms of fairness and are able to explicitly reason about such norms. However, they do not follow those norms in situations when sharing a resource results in less for themselves. We explored whether the gap between children’s actual behavior and their perceived norm (“moral dilemma”) might boost lying about the amount of donated stickers, but did not find statistical evidence. One drawback could be the categorical measure (i.e., 3, 4, 5 stickers), which may have masked a more nuanced difference around the median that could show on a continuous measure. Theoretically, it is possible that the donation and communication with the other children may have influenced children’s subsequent answers to the perceived norm question. Children who had lied about their donation might have intentionally lowered their answer in the perceived norm task in order to appear more prosocial. While counterbalancing the task order could alleviate these concerns, we were worried that asking the perceived norm question before the mini dictator game might heighten the relevance of prosocial sharing and increase the amount of donated stickers (and thus the relevance to lie).

A limitation of the current study is that a number of children (36%) failed to remember the amount of donated stickers in the memory check. On the one hand, this may suggest that they had difficulties with number representation. Research suggests that many children have difficulties with responding correctly to How-Many tasks even after they have counted an array correctly (Rittle-Johnson and Siegler, 1998; Sarnecka and Carey, 2008). Thus, a less number-based design might be more suitable to answer the research question. On the other hand, children may also choose to lie in the memory check to be consistent with the response to the test questions. Forty-two percent of children, who had lied in the test-questions, provided false information during the memory check.

The findings of this study shed light on social strategies underlying human cooperation. They suggest that social evaluation concerns develop in preschool years. Consequently, children simulate the perspective and evaluations of others and engage in strategic behavior to manage their reputation in interaction with peers (Tomasello, 2019). As a first step in investigating the emergence of lying for reputational concerns, the results suggest that 5-year-olds do not only lie to avoid punishment, or make others feel good, but that they begin to use lying as a strategy to manage the impressions others form of them and their group. This kind of lying for reputation management seems to emerge around the age of 5years. It is likely that lying for reputational concerns consolidates further with age, although moral norms may also partly prevent older children from whitewashing (Bussey, 1999). This research highlights that children do more than modifying their behavior in the presence of others to demonstrate that they are a good partner for collaboration (Leimgruber et al., 2012; Engelmann and Rapp, 2018, p. 2018). They intentionally modify others’ impressions of them on a mental level, independent of what they have actually done, through communication. From an evolutionary perspective, it seems likely that lying for reputational concerns is a recent achievement, which evolved in the context of coordinating with others to cooperate on a group level. Further data collection and comparisons with age groups should determine exactly how and when lying emerges ontogenetically as a strategy to manage reputational concern.

## Data Availability Statement

The raw data supporting the conclusions of this article will be made available by the authors, without undue reservation.

## Ethics Statement

The studies involving human participants were reviewed and approved by Ethikkommission der Fakultät für Psychologie und Bewegungswissenschaft der Universität Hamburg. Written informed consent to participate in this study was provided by the participants’ legal guardian/next of kin. Written informed consent was obtained from the minor(s)’ legal guardian/next of kin for the publication of any potentially identifiable images or data included in this article.

## Author Contributions

MK and UL contributed to conception and design of the study. MK organized the data collection, performed the statistical analysis, and wrote the first draft of the manuscript. UL wrote sections of the manuscript. All authors contributed to the article and approved the submitted version.

## Conflict of Interest

The authors declare that the research was conducted in the absence of any commercial or financial relationships that could be construed as a potential conflict of interest.

## Publisher’s Note

All claims expressed in this article are solely those of the authors and do not necessarily represent those of their affiliated organizations, or those of the publisher, the editors and the reviewers. Any product that may be evaluated in this article, or claim that may be made by its manufacturer, is not guaranteed or endorsed by the publisher.

## References

[ref1] Amemiya J. Liu Z. Compton B. J. Heyman G. D. (2020). Children’s judgements of positive claims people make about themselves. Infant Child Dev. 30:e2212. doi: 10.1002/icd.2212

[ref2] Bussey K. (1999). Children’s categorization and evaluation of different types of lies and truths. Child Dev. 70, 1338–1347. doi: 10.1111/1467-8624.00098

[ref3] Dunham Y. Baron A. S. Carey S. (2011). Consequences of “minimal” group affiliations in children. Child Dev. 82, 793–811. doi: 10.1111/j.1467-8624.2011.01577.x, PMID: 21413937 PMC3513287

[ref4] Engelmann J. M. Herrmann E. Tomasello M. (2012). Five-year olds, but not chimpanzees, attempt to manage their reputations. PLoS One 7:e48433. doi: 10.1371/journal.pone.0048433 23119015 PMC3485200

[ref5] Engelmann J. M. Over H. Herrmann E. Tomasello M. (2013). Young children care more about their reputation with ingroup members and potential reciprocators. Dev. Sci. 16, 952–958. doi: 10.1111/desc.12086, PMID: 24118719

[ref6] Engelmann J. M. Rapp D. J. (2018). The influence of reputational concerns on children’s prosociality. Curr. Opin. Psychol. 20, 92–95. doi: 10.1016/j.copsyc.2017.08.024 28858772

[ref7] Fu G. Evans A. D. Wang L. Lee K. (2008). Lying in the name of the collective good: a developmental study. Dev. Sci. 11, 495–503. doi: 10.1111/j.1467-7687.2008.00695.x, PMID: 18576957 PMC2570108

[ref8] Fu G. Heyman G. D. Cameron C. A. Lee K. (2016a). Learning to be unsung heroes: development of reputation management in two cultures. Child Dev. 87, 689–699. doi: 10.1111/cdev.12494, PMID: 27189397

[ref9] Fu G. Heyman G. D. Qian M. Guo T. Lee K. (2016b). Young children with a positive reputation to maintain are less likely to cheat. Dev. Sci. 19, 275–283. doi: 10.1111/desc.12304 25872952

[ref10] Gaertner L. Insko C. A. (2000). Intergroup discrimination in the minimal group paradigm: categorization, reciprocation, or fear? J. Pers. Soc. Psychol. 79, 77–94. doi: 10.1037/0022-3514.79.1.77, PMID: 10909879

[ref11] Herrmann E. Engelmann J. M. Tomasello M. (2019). Children engage in competitive altruism. J. Exp. Child Psychol. 179, 176–189. doi: 10.1016/j.jecp.2018.11.008, PMID: 30537568

[ref12] Heyman G. Barner D. Heumann J. Schenck L. (2014). Children’s sensitivity to ulterior motives when evaluating prosocial behavior. Cogn. Sci. 38, 683–700. doi: 10.1111/cogs.12089, PMID: 24069904

[ref13] Leimgruber K. L. Shaw A. Santos L. R. Olson K. R. (2012). Young children are more generous when others are aware of their actions. PLoS One 7:e48292. doi: 10.1371/journal.pone.0048292, PMID: 23133582 PMC3485147

[ref14] Mifune N. Hashimoto H. Yamagishi T. (2010). Altruism toward in-group members as a reputation mechanism. Evol. Hum. Behav. 31, 109–117. doi: 10.1016/j.evolhumbehav.2009.09.004

[ref15] Misch A. Over H. Carpenter M. (2016). I won’t tell: young children show loyalty to their group by keeping group secrets. J. Exp. Child Psychol. 142, 96–106. doi: 10.1016/j.jecp.2015.09.016, PMID: 26513328

[ref16] Misch A. Over H. Carpenter M. (2018). The Whistleblower’s dilemma in young children: when loyalty trumps other moral concerns. Front. Psychol. 9:250. doi: 10.3389/fpsyg.2018.00250, PMID: 29545763 PMC5839002

[ref17] Rapp D. J. Engelmann J. M. Herrmann E. Tomasello M. (2019). Young children’s reputational strategies in a peer group context. Dev. Psychol. 55, 329–336. doi: 10.1037/dev0000639, PMID: 30525833

[ref18] Rittle-Johnson B. Siegler R. S. (1998). “The relation between conceptual and procedural knowledge in learning mathematics: a review,” in The Development of Mathematical Skills. ed. Donlan C. (UK: Psychology Press/Taylor & Francis), 75–110.

[ref19] Sarnecka B. W. Carey S. (2008). How counting represents number: what children must learn and when they learn it. Cognition 108, 662–674. doi: 10.1016/j.cognition.2008.05.007, PMID: 18572155

[ref20] Smith C. E. Blake P. R. Harris P. L. (2013). I should but I won’t: why young children endorse norms of fair sharing but do not follow them. PLoS One 8:e59510. doi: 10.1371/journal.pone.0059510, PMID: 23527210 PMC3603928

[ref21] Sperber D. Baumard N. (2012). Moral reputation: an evolutionary and cognitive perspective. Mind Lang. 27, 495–518. doi: 10.1111/mila.12000

[ref22] Tajfel H. Billig M. G. Bundy R. P. Flament C. (1971). Social categorization and intergroup behaviour. Eur. J. Soc. Psychol. 1, 149–178. doi: 10.1002/ejsp.2420010202

[ref23] Talwar V. Crossman A. (2011). From little white lies to filthy liars. Adv. Child Dev. Behav. 40, 139–179. doi: 10.1016/B978-0-12-386491-8.00004-9, PMID: 21887961

[ref24] Tomasello M. (2019). Becoming Human: A Theory of Ontogeny. Cambrige, Mass: Harvard University Press.

[ref25] Warneken F. Tomasello M. (2008). Extrinsic rewards undermine altruistic tendencies in 20-month-olds. Dev. Psychol. 44, 1785–1788. doi: 10.1037/a0013860, PMID: 18999339

[ref26] Yamagishi T. Mifune N. (2008). Does shared group membership promote altruism?: fear, greed, and reputation. Ration. Soc. 20, 5–30. doi: 10.1177/1043463107085442

[ref27] Yazdi H. Heyman G. D. Barner D. (2020). Children are sensitive to reputation when giving to both ingroup and outgroup members. J. Exp. Child Psychol. 194:104814. doi: 10.1016/j.jecp.2020.104814, PMID: 32145479

[ref28] Zhao L. Heyman G. D. Chen L. Lee K. (2018). Telling young children they have a reputation for being smart promotes cheating. Dev. Sci. 21:e12585. doi: 10.1111/desc.12585, PMID: 28703471

